# Harmless or Threatening? Interpreting the Results of Molecular Diagnosis in the Context of Virus-Host Relationships

**DOI:** 10.3389/fmicb.2021.647730

**Published:** 2021-05-21

**Authors:** Fábio A. Abade dos Santos, Sara J. Portela, Teresa Nogueira, Carina L. Carvalho, Rita de Sousa, Margarida D. Duarte

**Affiliations:** ^1^National Institute for Agrarian and Veterinary Research, Oeiras, Portugal; ^2^Centre for Interdisciplinary Research in Animal Health (CIISA), Faculty of Veterinary Medicine, University of Lisbon, Lisbon, Portugal; ^3^Harrogate District Hospital NHS Foundation Trust, Harrogate, United Kingdom; ^4^Centre for Ecology, Evolution and Environmental Changes (cE3c), Faculdade de Ciências da Universidade de Lisboa, Lisbon, Portugal; ^5^National Institute of Health Doutor Ricardo Jorge (INSA), Lisbon, Portugal

**Keywords:** host-pathogen, detection, infection, commensalism, mutualism, molecular biology interpretation

## Abstract

Molecular methods, established in the 1980s, expanded and delivered tools for the detection of vestigial quantities of nucleic acids in biological samples. Nucleotide sequencing of these molecules reveals the identity of the organism it belongs to. However, the implications of such detection are often misinterpreted as pathogenic, even in the absence of corroborating clinical evidence. This is particularly significant in the field of virology where the concepts of commensalism, and other benign or neutral relationships, are still very new. In this manuscript, we review some fundamental microbiological concepts including commensalism, mutualism, pathogenicity, and infection, giving special emphasis to their application in virology, in order to clarify the difference between detection and infection. We also propose a system for the correct attribution of terminology in this context.

## Introduction

For centuries, the diagnosis of an infectious disease was solely based on clinical history and presentation. The first laboratorial technique used to visualise microbes was microscopy (Gest et al., 2004). By the 19th century, the relationship between disease and pathogens was established, triggering a cascade of microbiological lines of research during multiple epidemics such as smallpox, diphtheria, tuberculosis, cholera, among others. This was pioneered by Robert Koch, who formulated strict criteria to determine the cause-effect relationship between a microbe and a disease: (i) The microorganism must be found in diseased but not healthy individuals, (ii) the microorganism must be cultured from the diseased individual, (iii) the inoculation of a healthy individual with cultured microorganism must induce disease, and (iv) the microorganism re-isolated from the inoculated individual may match with the original. This clear set of rules has since become blurred with increasing understanding of the spectrum of relationships between an organism and its host, ranging from mutualism to parasitism.

The ambiguity regarding the microbe-host relationship has been further amplified following the massive explosion of alternative molecular detection methods, such as the polymerase chain reaction (PCR) (Mullis et al., 1992), which provided the scientific community with new revolutionary powerful tools to rapidly identify new organisms and genetic diseases. More recently, next generation sequencing (NGS) high throughput technology, which enables rapid sequencing of billions of DNA nucleotides, has enabled the study of microbiomes through the application of whole-genome sequencing (WGS) on microbial communities (metagenomics). The microbiome encompasses all the microorganisms living in or on any vertebrate animal, and can be sub-classified into the bacteriome, virome and mycobiome. The virome is composed of the collection of viruses that inhabit an organism (Lecuit and Eloit, 2013). In mammals, this includes viruses that infect the host, endogenous ancient virus-derived elements inserted in chromosomes, and viruses that infect members of the host’s microbiome, like the phages that replicate in bacteria (Virgin, 2014; Cadwell, 2015). Compared to bacteriomes, human and animal viromes are less well known. The study of viromes is hindered by several technical limitations, namely the lack of common markers for viruses, the huge heterogeneity of the virome components, the difficulties of working with small samples, the contamination of the samples by host DNA, the lack of adequate bioinformatic tools for analysis and the absence of robust, refined and updated databases (Zou et al., 2016).

Since then, methods such as DNA microarray and genome sequencing led to the detection of microbes whose pathogenic potential was unapparent (Young et al., 2015). Some unexpected viruses have been found in samples from both healthy and immunosuppressed patients without signs of overt disease (Sauvage et al., 2011; Kapusinszky et al., 2012). Others have even been found to have beneficial effects on human and animal health due to their ability to influence the structure and function of bacterial communities through prokaryotic viruses (Sandaa et al., 2018). The ability to detect a tremendous variety of viruses with unclear pathogenic potential (Table 1) has re-emphasised the importance of an accurate description of the symbiosis.

**TABLE 1:** Examples of commensal viruses.

Family	Evidence of colonisation
*Anelloviridae*	They are ubiquitous within the human species and have not yet been causally linked to any disease (Hino and Miyata, 2007; De Villiers and Zur Hausen, 2009). These viruses have been found in various organs, tissues and cell types (Tajiri et al., 2001; Thom et al., 2003), including plasma where they can cause persistent viraemia in 70% of worldwide population (Hino and Miyata, 2007)
*Papillomaviridae Polyomaviridae Circoviridae*	Three predominant families of viruses are found to colonise the human skin. Similarly to the skin microbiome, the skin human virome is composed of both resident and transient viruses (Lecuit and Eloit, 2013). High seropositivity (90%) to at least one human papilloma virus type has been reported in the human skin (Antonsson, 2012). Merkel cell polyomavirus, human polyomavirus 6, 7, and 9, are considered skin-tropic polyomaviruses existing chronically in healthy individuals (Feltkamp et al., 2013). Seropositivity of capsid protein VP1, a major structural component of the polyomavirus, can be detected in nearly 100% of the human population (Ott et al., 2000). Cyclovirus and other Circoviridae members are found in skin surface of both human and animals with cross-species transmission appearing possible (Li et al., 2011)
*Picornaviridae*	In the gastrointestinal tract, persistent or intermittent shedding of enteric viruses from healthy people is well established Human enterovirus Witsø et al., 2006 and parechovirus Olijve et al., 2018 are excreted by a large fraction of children under the age of five without any evidence of association with disease (Witsø et al., 2006; Olijve et al., 2018). Additionally, human enterovirus type 3 and 4 have the pig as reservoir Smith and Purdy, 2013
*Anelloviridae*	Kapusinszky et al. (2012) observed nearly constant shedding of anelloviruses including torque teno viruses Torque teno virus viraemia can be identified in nearly all individuals

Our perception of the role of viruses has shifted from solely sources of acute, persistent, or latent infections to commensal or even mutualistic organisms. The intricacy of virus-host relationships is reflected in the human genome composition, of which 5–8% is constituted by endogenous retroviruses (ERVs) (Nelson et al., 2004).

Today, molecular biology is an invaluable tool for diagnosis and research. However, the molecular detection of a potential pathogen in an animal or human can be easily misinterpreted. In the absence of corroborating clinical evidence, molecular detection often results in the assumption of an infection by default. This has become increasingly relevant in the field of virology, since the discovery of commensal and mutualistic viruses. The isolation and identification of a potential pathogen must be evaluated alongside the context of the microbial community to which it belongs to and the clinical evidence to suggest its interaction with the host.

In this manuscript, we review some fundamental microbiological concepts and explore how continuous discoveries in the field of microbiology demand some degree of re-framing of these concepts. We also discuss the strengths and weaknesses of several molecular diagnostic methods, exploring the differing implications of positive results. Finally, we propose a system for the correct attribution of terminology in this context.

## The Spectrum of Host-Organism Relationships

The initial definitions underlying most of the concepts of microbiology were largely pathogen-centred. Later, the recognition that many microbial agents may interact with certain hosts without causing disease led to the establishment of new terminology to describe the distinct situations where this might happen. Social relationships between organisms can be very complex. The same organism can engage in different types of biological relationships with other organisms or hosts and follow co-evolutionary paths.

Below, we describe some of the most common symbiotic relationships between microorganisms and their hosts in which at least one of the partners involved in the interaction benefits from this close relationship.

Symbiotic relationships between animals and microorganisms are common and well-known, although terms like commensalism, mutualism and parasitism, referring to different types of symbiotic relationships are sometimes misunderstood. In virology, these terms are largely underused.

### Commensalism

Commensalism describes the relationship between two organisms where one partner benefits whilst the other remains unaffected. Mutualism describes a relationship in which both partners take advantage (win-win relationship) and the term “parasitism” refers to the case where one partner takes advantage over the other (win-lose relationship). This is often applied when the invading organisms produce harm to the host – infection (Fierer et al., 2017).

One could argue that viruses are by nature intracellular parasites, given that they rely on the high-jacking of cellular processes to replicate. In fact, it was believed that the normal cellular function would be disturbed in this process, leading inevitably to emergence of disease (Griffiths, 1999). As such it is unsurprising that the term “commensal” was never used in the same way in virology (Mims et al., 1998). However, Griffiths (1999) proposed the concept of “commensal viruses,” suggesting they might remain within their host in a low replicative phase without therefore causing virus-induced cytolysis. For example, whilst an organism can be part of the natural and healthy microbiota without triggering any infectious disease, it can still pose the threat of pathogenicity. Threat as it can increase in number in the microbial community and lead to the onset of an infection. Because symbiotic relationships are dynamic and evolve over time, the imbalance of the bacterial diversity and load (dysbiosis) can become detrimental to the host (Zhang et al., 2015), and may promote opportunistic infections. Such is the case in the onset of pseudomembranous colitis following antibiotic therapeutic protocols, due to the overgrowth of the bacterial opportunistic pathogen *Clostridium difficile* in the human gut (David et al., 2019; Nogueira et al., 2019). Similarly, the overgrowth of the commensal yeast *Candida albicans* can result in oral thrush and oesophagitis (Deepa et al., 2014).

### Mutualism

Microbiota that reside in the epithelial tissue that is exposed to the external environment in the respiratory, gastrointestinal and vaginal tracts as well as in the skin since birth are often called commensal (Tlaskalová-hogenová et al., 2004), suggesting that neither it, nor its host, benefit or suffer from its presence. However, extensive research on the effect of microbiota on human and animal health has highlighted the presence of many symbiotic relationships between microorganisms and the host, generally beneficial to the host, and therefore better described as a type of mutualistic relationship (Macpherson and Mccoy, 2014).

The bacteria composing the human gut microbiota supply vitamins, aid in digestion of carbohydrates, maintain the integrity of mucosal barrier, and prevent overgrowth and invasion of pathogenic bacteria (Zhang et al., 2015).

Microbiota imbalances have therefore been linked to many human diseases including inflammatory bowel diseases, cardiovascular disease, obesity, and type 2 diabetes (Malinen et al., 2005; Frank et al., 2007; Turnbaugh et al., 2009; Larsen et al., 2010; Gerritsen et al., 2011; Kerckhoffs et al., 2011).

Mutualistic relationships between viruses and their hosts have also been revealed. Some authors have suggested that highly prevalent viruses, such as herpesviruses, may actually play a protective role against bacterial infection by boosting innate immunity (Dickinson, 2018). Some mouse herpesviruses, highly similar to the human Epstein Barr virus (EBV) and cytomegalovirus (CMV), activate the innate immune response and protect mice against bacteria (Barton et al., 2007). The γ-herpesvirus 68 (γHV68), for example, was found to protect against infection by *Listeria monocytogenes* and *Yersinia pestis* by sustaining IFN-γ production and macrophage activation (Barton et al., 2007). The murine norovirus can replace many of the benefits provided by commensal bacteria in the intestine (Kernbauer et al., 2014), and chronically it can lead to low expression of *Atg16L1*, an autophagy gene with allelic variants that predisposes to Crohn’s disease. The surprising finding that gyroviruses encode a protein that is specifically cytotoxic to cancer cells, raises the possibility that some viral infections can be beneficial in controlling the development of tumour cells (Los et al., 2009), whilst others are directly causative of certain kinds of cancer, such as Burkitt’s lymphoma and cervical cancer.

## Pathogenicity, Virulence, and Infection

A pathogen is a microorganism that can cause damage to its host. Pathogenicity results from the expression of virulent factors, proteins which are essential for the invasion and colonisation of the host, evasion of its immune system and nutrient uptake at its expense. Infection is the damage inflicted on the host during this process.

Casadevall and Pirofski (2000) highlighted that a coloniser organism can cause varying degrees of damage to its host, from none to substantial. The latter effect induces host responses that might be successful in eliminating the microbe or might be unsuccessful, consequently progressing to chronic infection. For those organisms that, once having colonised the host, induce no damage, its state is indistinguishable from “commensalism” (Casadevall and Pirofski, 1999).

SARS-CoV-2, is an example of a pathogen that induces a range of symptoms. Although a significant proportion of people infected with this virus do not display any symptoms (Yanes-Lane et al., 2020), detection of the virus in these individuals is always referred to as an infection, albeit asymptomatic, given the clear association between the pathogen and respiratory disease it is capable of causing. These asymptomatic infections differ from commensal colonisation due to the complete elimination of the offending organism by the immune system.

In 1999, Casadevall and Pirofski revised the term “pathogen” to mean a microbe capable of causing damage to its host, to highlight what they believed was the most relevant outcome of the host-pathogen interaction (Casadevall and Pirofski, 2000). Injury can result from either direct microbial action or the host immune response, or often both, can usually be identified through a combination of symptomatology, clinical examination and histology. However, damage being inflicted at a cellular level may escape detection by these methods. For example, high-risk human papillomavirus (HPV) types, responsible for the vast majority of cervical cancers, inactivate the essential tumour suppressor genes *pRb* and *p53* in host cells in order to induce in them a perpetual replicative state, necessary for optimal viral replication (Buitrago-pérez et al., 2009). Silencing of tumour suppressor genes in cervical cells does not immediate cause overt injury that is clinically or histologically identifiable. In fact, pre-cancerous dysplastic changes, can take a number of years to develop (Burd and Burd, 2003; Castle and Fetterman, 2009). Nonetheless, lack of evidence of damage does not indicate the offending organism is not a pathogen, when there is substantial reliable historical scientific evidence to the contrary.

Organisms can be described in terms of virulence and pathogenicity, which have been defined in various ways throughout the years, but overall describe the features or characteristics that enable an organism to cause disease and the degree or speed at which a pathogen can cause disease, respectively. Some concepts were recently revised by Casadevall and Pirofski (1999). These authors critically reviewed the origin and historical definitions of terms namely infection, commensalism, colonisation, persistence, infection, and disease and updated them in order to recognise current knowledge.

According to Lwoff (1957), “infection” is the introduction of a foreign entity that is capable of multiplying to produce additional infectious entities into an organism, regardless of whether this results in a disease. This definition encompasses the concept of subclinical or unapparent infections, which cause no signs or symptoms (Mahy, 2009).

Arguably, there are pitfalls to all definitions. Prions, infective proteins that can cause often devastating disease, might not technically be classified as microorganisms, and yet their pathogenicity is undeniable. This is also the case with infectious nucleic acid and infectious viral particles, which contain partial or complete viral genome. Some microorganisms pertaining to the healthy microbiota, although beneficial to their hosts much of the time, have the potential to cause disease though opportunistic infection. We rely on our microbiome to perform many human physiological functions, such as vitamin synthesis. Should these organisms be thought of as pathogens, as per Casadevall and Pirofski (1999), because they have the potential to cause damage? This seems to overlook their potential for beneficial effects. We do not agree with their revised definition of “infection,” we would argue that “acquisition of a microbe by host” should preferably be defined instead as “colonisation” when the impact on the host is unknown. After this process of “acquisition,” the pathophysiological sequence, it is often possible to predict and dependent on the pathotype – a group of organisms with same pathogenicity on a host. Only with evidence of pathogenesis caused by the colonisation of this organism, in an acute, chronic or intermittent manner, can the process be labelled an “infection.” This distinction better reflects the implications of this microbiological process in clinical practice.

## Inequality of Diagnostic Tests: Detection Versus Infection

There is a growing range of widely available diagnostic methods capable of detecting an organism or potential pathogen.

Culture-based methods provide evidence of viable infectious pathogens in the sample by demonstrating the growth of organisms *in vitro*. This is not only applicable to the growth of bacteria in culture media, but also to the growth of viruses and intracellular bacteria in susceptible eukaryotic cell lines. Although the growth of bacteria can be observed with the naked eye or simple light microscopy, growth of intracellular bacteria and viruses can be confirmed through staining techniques or identification of specific virus-induced cytopathic effects. Electron microscopy (EM) can be used to identify both mature and immature forms of viral particles within a cell. The simultaneous presence of both forms indicates active viral replication. However, it has a much lower sensitivity than molecular methods and requires specialised technicians and equipment.

Antigen tests detect certain proteins of a specific organism through immunoassays. They are quick and achieve a high specificity by targetting proteins or specific epitopes that are singular to the pathogen being detected. However, antigen tests can have low sensitivities, and therefore a higher rate of false negative results, when compared to PCR. Rapid influenza diagnostic tests (RIDTs), for example, which detect influenza virus nucleoproteins, only achieve a sensitivity of around 50–80% (CDC, 2020b). COVID-19 lateral flow rapid antigen tests also vary in sensitivity from 79%, when performed by laboratory scientists, to 58%, when performed by self-trained members of the public (Mahase, 2020). Rapid antigen tests for pathogens like group A beta-haemolytic streptococci and Hepatitis B virus, on the other hand, have substantially higher sensitivities of 90% and above (Joslyn et al., 1995). The sensitivity of these tests is dependent on factors such as timing of sample collection, collection technique and viral load (Tanei et al., 2014).

Importantly, a viable virus is not required for detection of an antigen. In infections like COVID-19 where viral shedding continues beyond the resolution of infection, meaning there is the presence of virus particles but no actual viable virus, a positive antigen test cannot distinguish between active infection with transmission potential and resolved infection without transmission potential (Cevik et al., 2021). Antigen detection should, therefore, be interpreted as current *or* recent infection and should not infer infectiousness.

Antibodies are produced by the humoural immune system in response to the detection of an antigen and can arise from either natural infection or vaccination. A positive antibody test thus indicates that there has been, at some point, an exposure to the organism.

There are several ways to distinguish between active and past infection. Detection of IgM antibodies, which are produced as the first response to a new infection but are only short-term and start dropping a few weeks after infection, is likely to indicate a current or recent infection.

IgG antibodies, on the other hand, are produced later, in the course of the infection, and can remain in the bloodstream for months to years. Because the lag time between initial infection and antibody production, timing of this diagnostic test is crucial to avoid false negative results.

The detection of specific class of antibodies against non-structural viral proteins is indicative of active viral replication and is therefore also a useful tool to detect ongoing infection. Unlike non-structural protein, which are present in much larger amounts, the structural proteins are fewer and less immunogenic. Their antibodies are thus short-lived and consequently un-detectable soon after the resolution of infection.

They have also been used in Differentiate Infected from Vaccinated Animals (DIVA) tests, given vaccines (with the exception of live and attenuated vaccines) do not result in the production of antibodies against non-structural proteins. This is the case with Hepatitis B. Surface antibodies can be produced in response to both active infection and as a result of vaccination, whereas core (non-structural) antibodies are only produced following natural infection.

Molecular based detection methods have some unquestionable advantages compared to the methods mentioned above, including their greater sensitivity, specificity and ability to be automated. However, the component that is being detected, whether that be genomic components or messenger RNA is paramount to the interpretation of a positive result.

Other group of molecular diagnostic tests detect genetic material that is specific an organism. The detection of mature messenger RNA provides evidence of active infection, as it implies gene expression, contrary to positivity by standard PCR, where detection may also represent the presence of nucleic acids from non-viable and therefore non-infectious organisms. The detection of mRNA can, therefore, help distinguish between viral latency and active replication (Lecuit and Eloit, 2013).

Although highly sensitive and specific, PCR test results are not always clear-cut with other interpretation issues such as the clinical significance of weaker signals, which represent a low copy number of a particular pathogen (Louie et al., 2000).

*In situ* hybridisation (ISH) detects viral genome in tissues or cells, by localisation of specific unique or repeated DNA and RNA sequences using complementary labelled probes. ISH demonstrates specific nucleic acid sequences in their cellular environment. As such, it can provide information regarding the level and place of mRNA expression, demonstrating the presence of newly synthesised viral DNA or RNA within cells, hence confirming the pathogens viability.

Some examples of diverse interpretations of molecular results have been provided over the years in both the bacteriology and virology fields. In 2019 the Centre for Disease Control and Prevention (CDC) alerted to the fact that although rapid molecular assays including PCR and other alternative nucleic acid amplification methods can detect viral RNA in respiratory specimens with high sensitivity and specificity, this result does not necessarily indicate detection of a viable virus or on-going influenza viral replication (CDC, 2019). Similarly, the detection of cytomegalovirus DNA from a patient’s serum cannot distinguish between active disease or latent infection (Ljungman et al., 2017), which are distinct situations from a clinical standpoint. Therefore, the use of sensitive laboratory techniques to test for the presence of novel viruses must be supported by additional clinical evidence to convincingly indicate that the detected virus was the cause of the observed disease (Griffiths, 1999).

The nuances of molecular biology result interpretation is a significant topic of discussion in the context of the recent global outbreak of severe acute respiratory syndrome coronavirus 2 (SARS-CoV-2) (Suri et al., 2020). Multiple studies have described a positive RT-PCR result several days after recovery (Lan et al., 2020; Young et al., 2020). Other cases have reported positive RT-PCR results following several consecutive negative results. The clinical and infection-control implications of this are unclear. However, the general consensus is that viral RNA detection does not necessarily indicate the presence of active infection or transmissible viable viral particles (CDC, 2020a). In fact, RT-PCR detects RNA, not an infectious agent, limiting the ability to determine the infectiousness of patients or animals with a positive PCR (Bullard et al., 2020). It has been suggested that quantifying viral loads may help to clarify the likely clinical picture. One study correlated the success of viral isolates with viral loads, and found that samples containing <10^6^ copies per ml never yielded an isolate. Thus it concluded that despite the detection of viral loads long after symptom resolution there would be little residual risk of infection (Wölfel et al., 2020). Given the general ambiguity around infective potential of recovered patients, future focus on detection of mRNA throughout the course of the disease, rather than RNA, might produce more elucidating results. This COVID-19 pandemic has highlighted the importance of correct interpretation of molecular biology test results given its potential to influence global guidance on appropriate time of patient discharge and isolation length.

## Conclusion

Molecular biology, biotechnology, genomics, and bioinformatics were the basis for one of the most important revolutions in recent microbiology, providing a boom of different conceptual methods that quickly replaced most of the classic, time-consuming, and laborious laboratorial techniques used for the diagnosis of microbiological diseases. This revolution represented a change of focus from the agent itself to the simple identification of nucleic acids. However, contrary to the method of culture and isolation in cell lines, the detection of nucleic acid does not necessarily indicate the presence of viable organisms capable of replication and infection, given viral particles can persist even after resolution of infection. Other methods aimed at detecting messenger RNA or non-structural proteins on the other hand can reliably indicate an active infection.

Molecular biology is not only enabling the identification of new viruses but also the genotyping and viral load quantification of these organisms. However, virology has lagged in the exploration of the different types of virus-host relationships. For viruses, whose pathogenic virulence is recognised, the characterisation of virus-host relationships is simple. Nonetheless, a growing number of non-pathogenic viruses establish states of commensalism or mutualism with their hosts. At times, key features of these complex microbiological states and processes overlap, impairing recognition and classification. This issue is further exacerbated by the nature of most investigations, which are not longitudinal and therefore cannot capture such dynamics.

**FIGURE 1:**
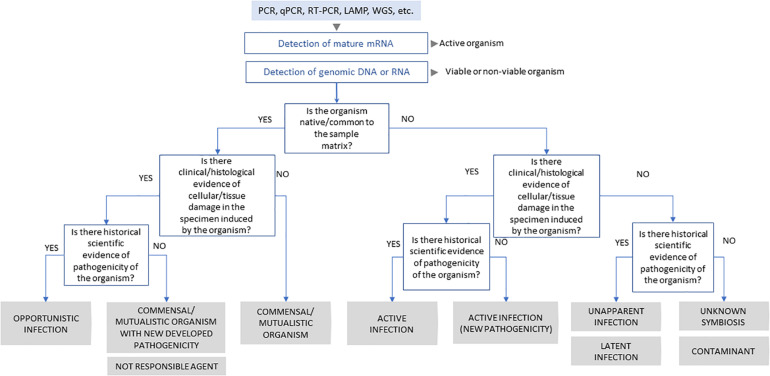
Schematic representation of scientific process to classification of virus-host interaction.

Thus, we emphasise the importance of combining physiopathological evidence to the molecular data when describing the novel presence of a microorganism in a host. Where this is not possible, we suggest the use of more conservative language which avoids charged terms such as “infection” and “pathogen.” To aid the appropriate use of what should be standardised terminology, we propose a rationale to characterise microbe-host relationships (Figure 1).

## Author Contributions

FAAS, SP, and TN: conceptualisation and writing – original draft preparation. CC: writing – original draft preparation. RS: writing – review and editing. MD: conceptualisation and writing – review and editing. All authors contributed to the article and approved the submitted version.

## Conflict of Interest

The authors declare that the research was conducted in the absence of any commercial or financial relationships that could be construed as a potential conflict of interest.
